# Spatial Patterns of *Frangula alnus* (Rosales: Rhamnaceae): Implications for Invasive Plant Management

**DOI:** 10.3390/biology12111393

**Published:** 2023-11-01

**Authors:** Jennifer Greenleaf, Roghaiyeh Karimzadeh, Yong-Lak Park

**Affiliations:** 1 Division of Plant and Soil Sciences, West Virginia University, Morgantown, WV 26506, USA; jennifers.greenleaf@gmail.com (J.G.); roghaiyeh.karimzadeh@mail.wvu.edu (R.K.); 2 Department of Plant Protection, Faculty of Agriculture, University of Tabriz, Tabriz 5166614888, Iran

**Keywords:** *Frangula alnus*, glossy buckthorn, invasive species, spatial pattern, distribution, SADIE, geostatistics

## Abstract

**Simple Summary:**

Glossy buckthorn (*Frangula alnus*) (Rosales: Rhamnaceae) is an invasive shrub from Europe, threatening native vegetation in open and disturbed habitats. To determine the spatial distribution patterns of glossy buckthorn, the density and presence of *F. alnus* were recorded in 1412 transect sample points located on four sites in the Allegheny National Forest, Pennsylvania, USA. Geostatistical analyses were used to determine spatial autocorrelation among individual *F. alnus* samples and to measure their spatial dependency. Spatial patterns were examined using spatial analyses by distance indices (SADIE), and buffer analyses were conducted to determine the relationships between proximity to unpaved forest roads and both *F. alnus* density and presence. The results of this study showed that the density and presence of *F. alnus* decreased as the distance from the forest road increased. We also found a significant spatial aggregation of *F. alnus* in all four study sites. These findings imply that spatially targeted management of *F. alnus* would be possible by locating the patches of *F. alnus* and applying control measures only where needed.

**Abstract:**

Glossy buckthorn (*Frangula alnus*) (Rosales: Rhamnaceae) is an invasive shrub from Europe that has been invading North America for over a century and threatening native vegetation in open and disturbed habitats. The treatment of *F. alnus* is currently restricted to the roadside, suggesting any individual *F. alnus* residing within the forest would be left unmanaged and would continue to spread in the area. This research was conducted to determine the spatial patterns and relationship of *F. alnus* with forest roads. The presence and density of *F. alnus* at 1412 sample points were recorded on four sites in the Allegheny National Forest, Pennsylvania, USA. Buffer analyses were conducted along roads to determine the relationship between *F. alnus* density and proximity to forest roads. Geostatistics and spatial analysis by distance indices (SADIE) were used to characterize the spatial pattern of *F. alnus*. Results of this study showed that *F. alnus* was spatially aggregated and resided beyond forest roads. Both the density and presence of *F. alnus* decreased as the distance from the forest road increased. These results imply the potential for precision management of *F. alnus* by locating and managing only where *F. alnus* presents.

## 1. Introduction

Glossy buckthorn (*Frangula alnus*) (Rosales: Rhamnaceae) is an invasive shrub from Europe and has been invading North America for over a century [1]. In Europe, *F. alnus* was historically used to create superior charcoal for gunpowder production, is used as an antibacterial and antioxidant medicine [2], and plays a natural role in the ecosystem where it has coevolved with other wildlife [3]. In the late 1800s, it was intentionally introduced as an ornamental shrub to Canada. It then escaped cultivation and was first discovered growing wild in London, Canada in 1898 [4]. Since then, *F. alnus* has spread throughout Southwestern Canada and the Northeastern USA, proving itself to be an aggressive pest as it forms dense monospecific patches in disturbed areas, along roads, and throughout fields and clearings [5]. As of 2023, 29 states in the USA harbor this invasive plant [6].

Multiple factors contribute to the success of *F. alnus* in North America [7,8,9]. It appears to lack the natural herbivores that it experienced in Europe. Our observations of *F. alnus* populations in Belgium and Germany have noted that *F. alnus* are often small and sparse and that the leaves often show signs of pathogens and insect herbivory. In contrast, in the USA, *F. alnus* forms large, dense patches and the leaves appear to be largely undamaged. Known herbivores such as *Zygina suavis* (Hemiptera: Cicadellidae) and *Gonopteryx rhamni* (Lepidoptera: Pieridae) [10,11] could limit the growth and reproduction of *F. alnus* in its native habitats, but these specialist herbivores are not known to exist in North America.

One of the major regions in the USA where *F. alnus* invaded and established very successfully is the Allegheny National Forest, Pennsylvania, USA. The presence of *F. alnus* in the Allegheny National Forest was first reported in 1989. At that time, local forest management favored *F. alnus* due to timber harvests creating open canopies. Traditionally, detection of *F. alnus* is done by roadside surveys, stand exams, and surveys of areas marked to receive timber management. In 2007, the USDA Forest Service conducted an initial survey of *F. alnus* and a closely related species *Rhamnus cathartica* (Rosales: Rhamnaceae) in the Allegheny National Forest [12]. The survey area covered 1420 ha, and only 20 ha did not have either buckthorn species, and 55% of the survey area had a moderate or heavy infestation. Light feeding damage was also noted due to deer browsing, but it was also mentioned that this seemed to not affect invasion [12].

According to the Forest Service database entry for *F. alnus* in the Fire Effects Information System (https://www.fs.usda.gov/database/feis/plants/shrub/fraaln/all.html, accessed on 20 August 2023), the most effective method of control may be to target seedlings and to stop new invasions from occurring. Reducing disturbances is also recommended because *F. alnus* responds positively to openings and disturbances [13] although the complete eradication of *F. alnus* has historically not been successful [14,15]. According to Converse [16] and Schuster et al. [17], *F. alnus* may be managed by cutting, mowing, girdling, excavating, burning, and underplanting. Herbicide treatment of girdled stems and cut stumps, prescribed burns followed by foliar herbicide, and the removal of roots from cut areas also can be used to control *F. alnus* [18]. Previous studies [17,19] also suggested the complete removal of *F. alnus* for initial 2-year management to prevent its reinvasion. In the Allegheny National Forest, foliar herbicides (e.g., glyphosate), pre-emergent herbicides (e.g., sulfometuron methyl), and manual cutting or mastication are used to control *F. alnus* [20]. However, these practices of detection and treatment have been used for *F. alnus* only alongside forest roads where they are visually detectable, although *F. alnus* could be growing inside the forest far from the road. This indicates that any individual *F. alnus* residing within the forest would be left unmanaged and would continue to spread in the area. To maximize the management of *F. alnus*, it is vital to understand its spatial distribution patterns and how it is associated with roadways in the forest.

Determining the spatial distribution pattern of invasive plants is one of the fundamental measures providing important information about their population dynamics and dispersal ecology. Subsequently, visualizing the spatial distribution of the invasive plants on a map would be helpful for their population management [21]. Geostatistics is a statistical procedure that uses sample values and locations simultaneously to analyze spatial dependency and to estimate values at unsampled locations (i.e., interpolation) [22]. To quantify spatial dependency, the semivariance of paired samples is plotted against the lag or *h*, the separation distance between sample pairs (i.e., semivariogram) [22]. Geostatistics employs an interpolation method, called kriging, by using the spatial structure of data (i.e., semivariogram) to estimate values at unsampled locations [22]. Although semivariograms determine the spatial structure and spatial autocorrelation, they do not statistically determine the spatial distribution patterns such as aggregation, random, or uniform. Spatial analysis by distance indices (SADIE) is a methodology explicitly developed for determining spatial patterns by analyzing spatial count data [23]. In SADIE, the spatial pattern of samples is determined by calculating the index of aggregation (*I*_a_) based on the distance to regularity [23], the minimum effort that the individuals in a sample would need to expend to move to an arrangement where there was an equal number in each sample unit. In addition, SADIE can be used to generate a clustering map, called a red-blue map, showing patches (i.e., spatial clusters of significantly higher count) or gaps (i.e., spatial clusters of significantly lower count). Detailed descriptions of this method and its applications can be found in published articles [23,24,25]. Geostatistics and SADIE together could be a novel way to determine the spatial structure of *F. alnus,* statistically test spatial aggregation, and generate interpolated maps of *F. alnus* distribution which can be used for developing a new strategy for management.

The objectives of this study were to determine (1) the relationship of *F. alnus* distribution with the proximity to forest roads and (2) the spatial distribution patterns of *F. alnus* using advanced geostatistical methods. Understanding the spatial distribution patterns and relationship of *F. alnus* with forest roads could increase both the detection and the successful treatment of *F. alnus*.

## 2. Materials and Methods

### 2.1. Study Sites

**Figure 1:**
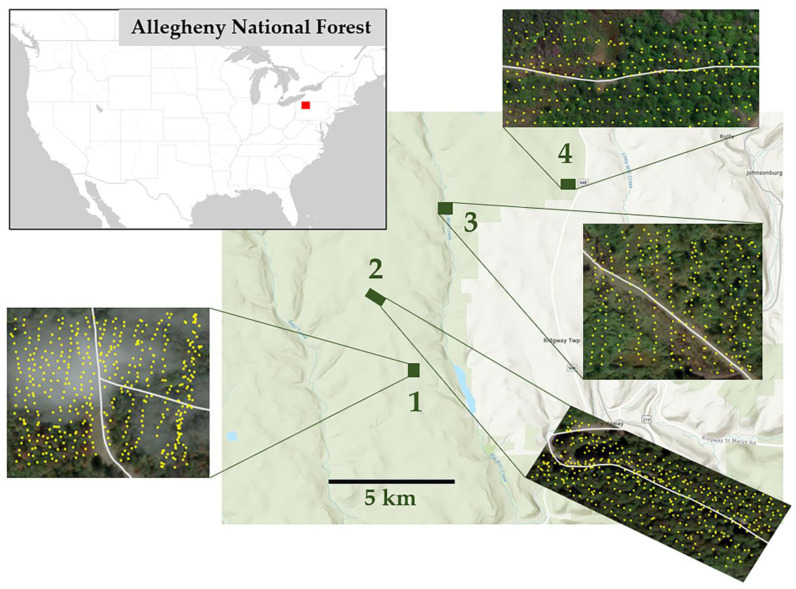
The location and sample layout for each (2.5 ha) of the four research sites in the Allegheny National Forest, Pennsylvania, USA: site 1 (41.43704, −78.79137); site 2 (41.45674, −78.80247); site 3 (41.47875, −78.77288); site 4 (41.485216, −78.741848). Field surveys were conducted in 2021 and 2022. Yellow dots in each map indicate sample points where a measurement of *F. alnus* was taken, and white lines indicate unpaved forest roads.

This study was conducted in the Allegheny National Forest near the borough of Ridgway, Pennsylvania, USA in 2021–2022. Four 2.5-ha sites with known infestations of *F. alnus* were selected for this study (Figure 1). The geocoordinates of the center of the four sites are as follows: site 1 (41.43704, −78.79137); site 2 (41.45674, −78.80247); site 3 (41.47875, −78.77288); site 4 (41.485216, −78.741848). The Allegheny National Forest is composed mostly of even-aged mature hardwood forests with closed canopies. Sites 1 and 2 consist of deciduous forests and unpaved forest roads. Site 3 is split down the middle by a powerline with an unpaved road and includes patches of grasslands and a deciduous forest. Site 4 is split by an unpaved road; on one half is a deciduous forest, and on the other a patch of coniferous trees and marshes.

### 2.2. Sampling Method

A total of 1412 sample points were placed every ca. 8 m on transects in four sites. Transects ran parallel to the longest borders of each site and also ran roughly parallel to roads within the sites. At each sample point, geo-coordinates and *F. alnus* data were recorded by using Avenza Maps version 4.2.2 (Avenza Systems Inc., Toronto, ON, Canada) and a GPS (Garmin GPSMAP^®^ 64st, Garmin Ltd., Olathe, KS, USA).

Because many *F. alnus* inside the forest were small and short, it was impossible to take measurements of diameter at breast height (*DBH*). Therefore, we developed an index (called Buckthorn Index) that can include the measurement of small and short *F. alnus* equivalent to *DBH*. The Buckthorn Index (*BI*) was measured in the following manner. Within a circular area of 2.2 m^2^ at each sample point, all *F. alnus* individuals were given a value based on *DBH* and the number of *F. alnus*, but instead of measuring *DBH* directly, values were estimated by a point system. Each *F. alnus* within the 2.2-m^2^ area at each sample point that also reaches breast height was given one *BI* point for every 5 mm of stem diameter. Any *F. alnus* individuals that were too short to reach breast height were given a value of 1; this allowed for the inclusion of the many *F. alnus* saplings present in this study. The scores of *BI* values of all *F. alnus* found at each sample point were summed to represent the *BI* value for the sample point. Data from Avenza Maps was exported as a shapefile. Data from the Garmin GPS was exported to DNR GPS (Minnesota DNR, Saint Paul, MN, USA) and was then converted into a shapefile. Both shapefiles were combined in ArcGIS Pro Version 2.8.0 (Environmental Systems Research Institute, Redlands, CA, USA).

### 2.3. Buffer Analysis with GIS

The georeferenced *BI* data and site boundary shapefiles were uploaded to ArcGIS Pro. Shapefiles were created to designate all roads within and surrounding the four sites. Buffers were added site by site to the road shapefiles in increments of 5 m until all data points were within a buffer that represented a specific range of distance from the road (e.g., 0–5 m, 5–10 m, and so on). Regression analysis [26] was used to determine the relationship between *BI* values and the distance from the road. This buffer analysis was also done with the presence dataset (i.e., presence/absence of *F. alnus* at each sample point).

### 2.4. Geostatistical Analysis

To determine the spatial dependency of *F. alnus* distribution at each site, semivariograms were created for both the *BI* data and presence data. Semivariance was defined by the following equation [22]:

$$\gamma \left(h\right)=\frac{1}{2N\left(h\right)}\sum_{i=1}^{N\left(h\right)}{\left[Z\left(x_{i}\right)-Z\left(x_{i+h}\right)\right]}^{2}$$

where γ(*h*) is semivariance at distance between sample pairs *h*, *N*(*h*) is the number of sample pairs separated by lag distance *h*, and *Z*(*x_i_*) and *Z*(*x_i_* + *h*) are derived from sample values at point *i*. Active lag distances were chosen based on optimizing the lowest residual sum of squares (RSS) and highest R^2^ values. The uniform lag class distance intervals were between 7–9 m depending on the site. Three semivariogram parameters were used to describe the spatial structure of *F. alnus* distribution: range (*A*_0_, maximum distance at which two points are spatially correlated), sill (semivariance value at the range), partial sill (*C*, the difference between sill and nugget), and nugget (*C*_0_, *y-intercept*) [22].

The degree of spatial dependency (*DSD*) was measured by using the following formula [27]:

$$DSD=\left(\frac{C}{C_{0}+C}\right)\times 100$$

Spatial dependency is considered weak when *DSD* < 25%, moderate when 26% < *DSD* < 75%, and strong when *DSD* > 76% [27]. The assumption that each lag class had more than 30 sample pairs was met, and eight analyses (4 sites, each with *BI* and presence values of *F. alnus*) were conducted.

Based on the assumption that forest roads would affect the distribution patterns of *F. alnus*, current detection and management have been focused on *F. alnus* only alongside forest roads. In such a case, the data would exhibit anisotropy (i.e., directional effect). Anisotropy was tested by constructing semivariograms in four directions (0, 45, 90, and 135°) with a 22.5° offset tolerance [22]. The anisotropy factor (i.e., the quotient between the minor and major axes of anisotropy) was used to determine the existence of directional effects. Where the anisotropy factor was <0.5, anisotropy would be used in the analyses [28].

Once semivariogram models were developed, the parameters were used for estimating *BI* and presence values at unsampled locations by using kriging (i.e., interpolation to create distribution maps). Cross-validation was used for assessing the precision of predictions and selecting the best model [28]. The models with mean prediction error close to zero, mean squared deviation ratio close to 1, and smallest root-mean-square prediction error were selected to generate distribution maps by using ArcGIS Pro. All geostatistical analyses were conducted using GS+ Version 9.0 (GammaDesign Software, Plainwell, MI, USA).

### 2.5. Spatial Analysis by Distance Indices (SADIE)

Spatial patterns of *BI* and the presence of *F. alnus* were investigated by calculating the index of aggregation (*I*_a_) based on the distance to regularity. A value of overall *I*_a_ = 1 indicates a random spatial distribution; *I*_a_ > 1 indicates aggregation of observed counts; and *I*_a_ < 1 indicates a uniform spatial pattern. The statistical significance of spatial aggregation was determined by *P*_a_ values, the probability level of *I*_a_. The *P*_a_ < 0.05 indicated that spatial aggregation was statistically significant. Clustering indices ($v_{i}$ or $v_{j}$) were defined for each sample point to determine whether it belonged to a patch (significantly higher than the mean) or a gap (significantly lower than the mean). Overall spatial clustering was determined by $\bar{v_{i}}$ (the mean value of $v_{i}$ of all patches) and $\bar{v_{j}}$ (the mean value of $v_{i}$ of all gaps) values and their associated probabilities, *P*$\bar{v_{i}}$ and *P*$\bar{v_{j}}$, respectively. Values of $v_{i}$ > 1.5 indicated that the sample point is within a patch and values of $v_{j}$ < −1.5 showed that the sample point is within a gap. Values of *P*$\bar{v_{i}}$ < 0.05 and *P*$\bar{v_{j}}\;$< 0.05 indicated significant clustering into patches and gaps, respectively [23,29]. The analyses were conducted by using SADIEShell Version 2.0. The $v_{i}$ and $v_{j}$ values were used to produce red-blue maps and determine the locations of patches and gaps [24]. The maps were generated using the inverse distance weighted interpolation method in ArcGIS Pro.

## 3. Results

The average *BI* values of *F. alnus* in all four sites were 16.83 ± 4.28 (SEM), and maximum *BI* values at sites 1, 2, 3, and 4 were 82, 43, 109, and 105, respectively. Approximately 75% of sample points had at least one *F. alnus* and site 3 had the highest *BI* values (Table 1).

### 3.1. Analysis

**Figure 2:**
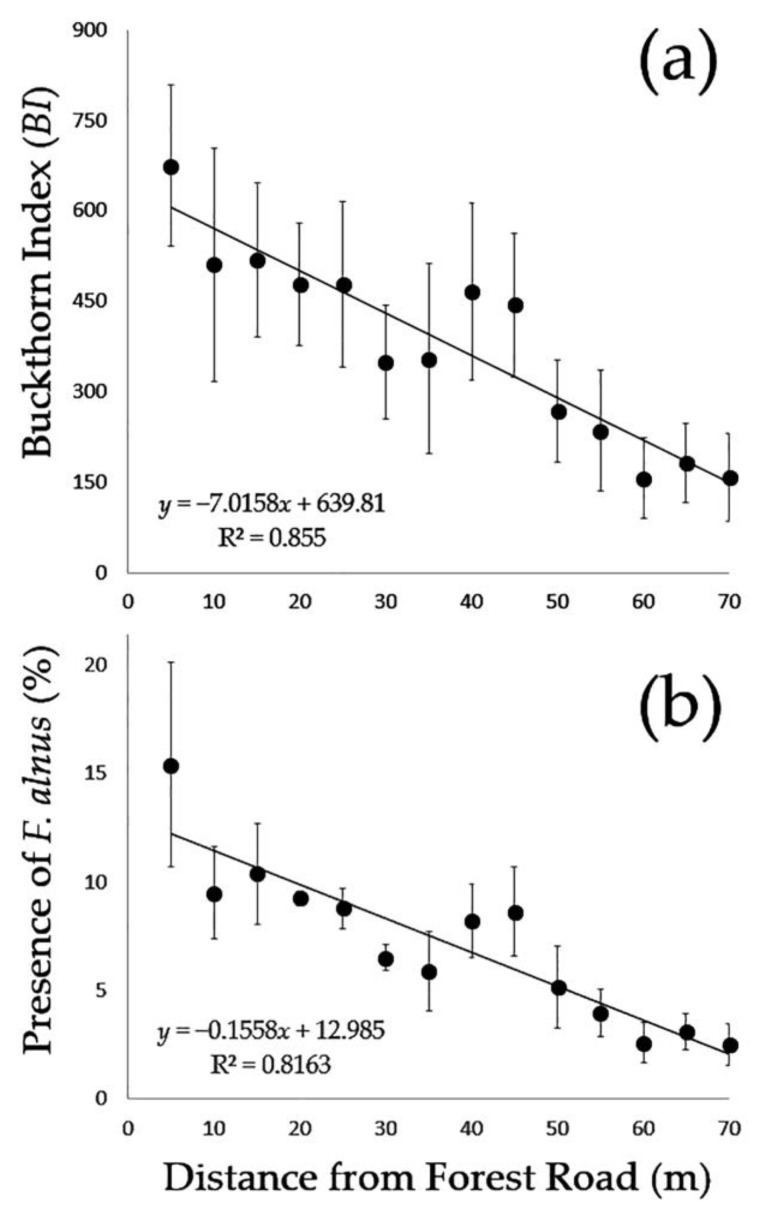
Relationships of Buckthorn Index (*BI*) (**a**) and percent presence of *F. alnus* (**b**) with the distance from the forest road.

The results of the buffer analysis and regression analysis showed that the presence of *F. alnus* (*F* = 51.29; d.f. = 1, 11; *p* < 0.001) and *BI* values (*F* = 49.75; d.f. = 1, 11; *p* < 0.001) decreased as the distance from the road increases (Figure 2). This indicates a higher density and presence of *F. alnus* closer to the forest road, but *F. alnus* still resided within the forest far from the forest road.

### 3.2. Geostatistical Analysis

Anisotropic semivariograms of both *BI* and presence values showed very low directional variations with an anisotropic factor > 0.5 for all datasets (Table S1). Therefore, we generated isotropic semivariograms for both *BI* and presence values. The semivariograms of presence values for *F. alnus* indicate that sites 1 and 4 fit exponential models, site 2 fit a linear model, and site 3 fit a nugget model. The ranges of presence values ranged from 4 to 204 m. *DSD* ranged from 0 to 87.94%; site 3 had a weak spatial dependency while sites 2 and 4 had a moderate spatial dependency, and site 1 had strong spatial dependency. The semivariograms of *BI* values (Table 2) indicate that site 1 fitted a spherical model while sites 2, 3, and 4 fit exponential models. These models indicate the presence of spatial dependency or spatial autocorrelation. The ranges of *BI* value ranged from 31 to 341 m, the distance of spatial dependency between two sample points. *DSD* ranged from 52.3–70.0%, indicating that all four sites exhibited moderate spatial dependency.

**Figure 3:**
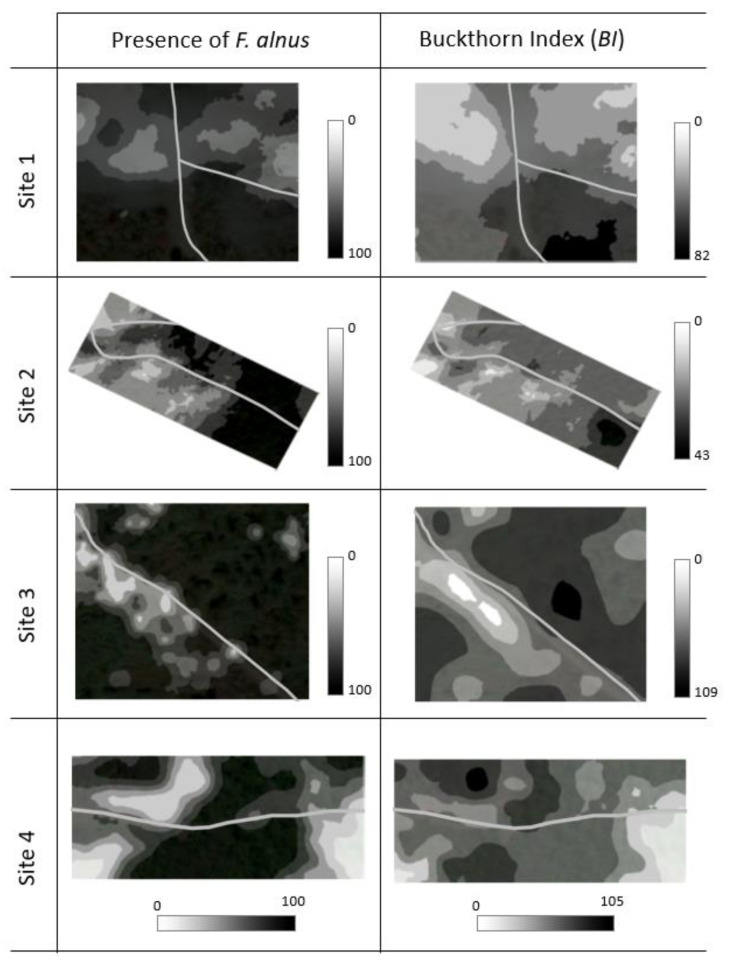
Interpolation maps for the presence of *F. alnus* (%) and Buckthorn Index (*BI*). The maps were generated by using kriging. Gray lines indicate forest roads.

Interpolated maps of *BI* values by using kriging revealed that sites 1, 2, and 3 showed apparent positive associations of *F. alnus* density with proximity to the forest road (Figure 3). Only site 3 showed an apparent association with proximity to the forest road when the presence of *F. alnus* was mapped.

### 3.3. SADIE

**Figure 4:**
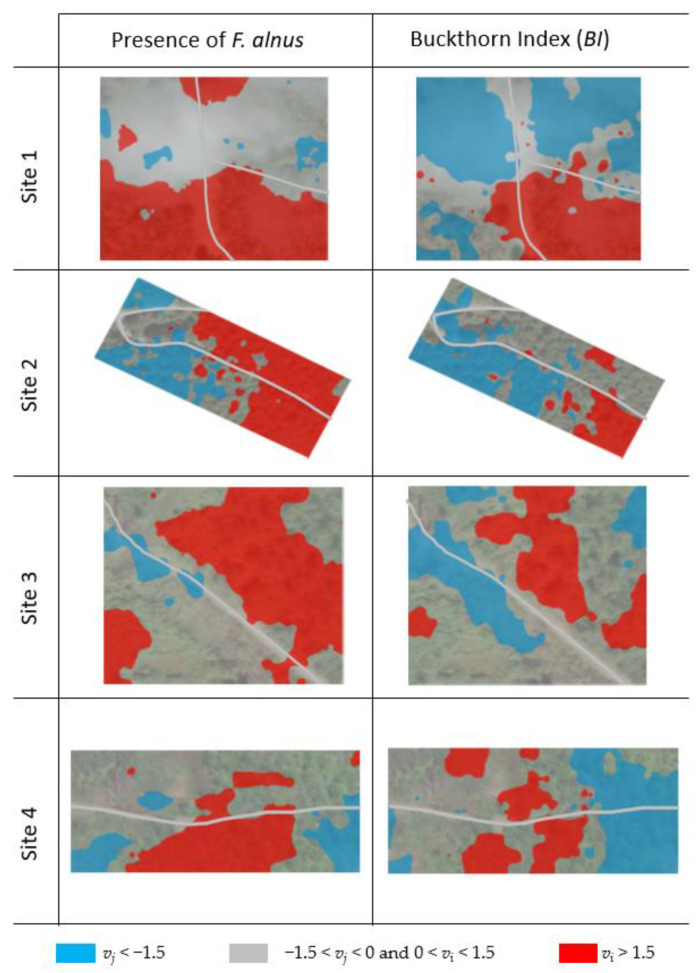
Clustering maps for the presence of *F. alnus* (%) and Buckthorn Index (*BI*). Red and blue areas in cluster maps indicate patches and gaps, respectively. Blue areas represent “patches” where *v_i_* > 1.5 while red areas represent “gaps” where *v_j_* < −1.5. Gray lines indicate forest roads.

The SADIE results of both *BI* and presence values on all four sites revealed significant spatial aggregations (i.e., *I*_a_ > 1 and *P*_a_ < 0.01) (Table 3). Eight red-blue maps derived from *v_i_* and *v_j_* values were generated using inverse distance weighted interpolation (Figure 4). No obvious correlation between *BI* values and the road was found in sites 1 and 4 while sites 2 and 3 showed obvious spatial associations of the presence of *F. alnus* with the roads.

## 4. Discussion

**Figure 5:**
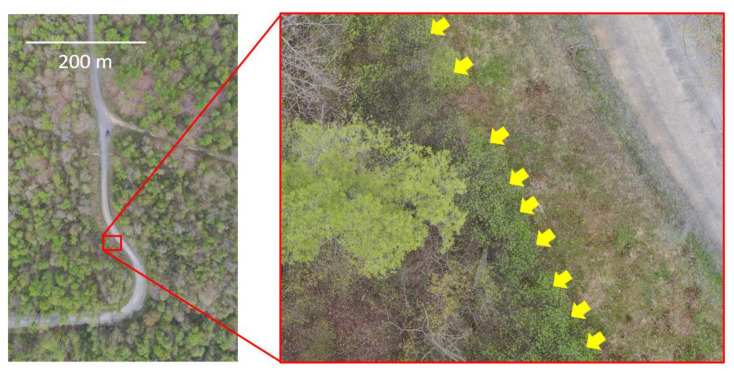
Aerial view of linear infestations of *F. alnus* along the forest road. Yellow arrows indicate *F. alnus*. The aerial photos were taken by a drone (DJI Mavic 2 Pro, SZ DJI Technology Co., Ltd., Shenzhen, China).

Although *F. alnus* has invaded North America since the late 1800s, this is the first study to determine the spatial patterns of *F. alnus* in North America. In Europe, the only study that examined the spatial structure of *F. alnus* distribution was conducted by Mosner et al. [30] who investigated the population genetic structure of *F. alnus* by relating patterns of genetic diversity with spatial distribution across Germany. Their spatial analyses showed significant spatial dependency or autocorrelation between individuals in an *F. alnus* population, but no relationship existed between environmental factors such as temperature and precipitation and the genetic distribution of *F. alnus*. The results of our study conducted at a much finer spatial scale showed that *F. alnus* exhibited spatial dependency and significant spatial aggregation, according to semivariograms and SADIE, respectively. In addition, our field observations noted that there were often thick patches of *F. alnus* growing in a thin line (approximately 1–3 m in thickness) on the edges of many forest roads and clearings (Figure 5). These linear patches of *F. alnus* are currently subject to control with mechanical control followed by herbicide application in the Allegheny National Forest where this study was conducted.

*F. alnus* in North America has a strong competitive advantage over native understory vegetation due to the lack of natural enemies, vegetative spread from underground runners, and tolerance to a wide range of soil conditions [10,11,31,32,33]. *F. alnus* aggressively outcompetes native regrowth by allelopathy and creates dense patches that inhibit any other understory plants from growing [5,32,33]. In addition, *F. alnus* maintains the unique ability to both leaf out before other deciduous trees and to retain its leaves longer into the fall than other deciduous trees [31]. These ecological characteristics equate to a longer growing season, ultimately enabling this invasive shrub to take over vital resources and crowd out native undergrowth with its vigorous maturation. *F. alnus* invades by taking advantage of gaps in forest canopies and along roadways. Burnham and Lee [34] found that *F. alnus* was 96 times more abundant in logged areas than in uncut plots and that forest disturbances benefit its growth and survival. Cunard and Lee [35] found that *F. alnus* loses its competitive advantage when surrounding shade-tolerant trees have greater basal areas and when sunlight is limited. They also suggested that *F. alnus* could be controlled by simply letting the forest advance to older successions; however, this would not work alongside roads where vegetation is regularly cleared to make room for vehicles in the forest. Therefore, we anticipated that *F. alnus* would exist in higher densities alongside the disturbed roadways, and falter under the forest canopy because *F. alnus* dispersion could be facilitated by vehicles and other machinery, or that birds may be utilizing the canopy gaps generated by roads to access *F. alnus*. The results of our study indicate that *F. alnus* density and its presence decrease as the distance from the road increases in the forest. Because the current management of *F. alnus* is limited to the side of the road where these invasive shrubs can be spotted from within a vehicle, the current method for *F. alnus* management may not be sufficient, given there are individual *F. alnus* outside the visible range from the forest road. We have found that many of them have successfully invaded under the tree canopies inside the forest.

Environmental factors such as edaphic and topographic factors and types of surrounding vegetation can influence the distribution pattern of *F. alnus*. Our study sites included habitats with both deciduous and coniferous trees, and we did not find their spatial relationship with *F. alnus* except forest roads. However, we noticed that *F. alnus* within the forest canopy and away from the road had fewer fruits, though many were >1 m tall. Future studies may seek to quantify this difference, with the importance being that if these individuals are not reproductive, they would be less of a threat and therefore less of a priority for treatment. It should be noted that the distribution of a plant is reliant not only on local environmental factors such as soil nutrient conditions and climate [7,9] but also on the species’ vegetative spread and seed dispersal methods. The seeds of *F. alnus* are typically bird-distributed, and it is reasonable to believe that a bird could travel between all four sites with the fruit; this would suggest correlations of both the genetics and the distribution of *F. alnus* between sites.

Based on our findings in this study, there are three major implications for *F. alnus* management. Firstly, although a higher density of *F. alnus* was found near forest roads, there still are patches of *F. alnus* inside the forest or hard-to-access areas where field surveyors generally do not check. Finding and managing such remote populations of *F. alnus* would prevent the spread and re-invasion of *F. alnus* after a successful control [19]. Current drone technologies including sensors and high-resolution satellite imagery could help find *F. alnus* located remotely or in hard-to-access areas [21]. Secondly, the strong spatial aggregation of *F. alnus* revealed from our study implies the possibility of spatially targeted invasive plant management. By detecting patches of *F. alnus* both roadside and inside the forest, control measures can be applied site-specifically. Such site-specific pest management generally increases pest management efficiency and reduces the number of herbicide inputs, application costs, and negative effects on surrounding wildlife [36]. Spatial distribution maps such as the ones generated in this study could be used to set management zones for site-specific pest management [36]. Lastly, the results of this research provide recommendations for how far from the road the control measures (such as herbicide, mechanical removal, or both) need to be applied to meet a certain goal under the allowable resources. We suggest treatments reach approximately 25, 60, and 70 m into the forest from the road to obtain goals of 50%, 90%, and 95% of *F. alnus* control, respectively, based on our regression analysis (Figure 2).

## 5. Conclusions

This is the first exploration of the spatial patterns of *F. alnus* on an individual plant scale in North America. Using advanced geostatistical methods (i.e., semivariograms and SADIE together), our study showed negative spatial associations of density and presence of *F. alnus* with the proximity to forest roads while the management of *F. alnus* is currently limited to what can be easily seen from the road. This information can improve the success and efficiency of *F. alnus* management. Monitoring efforts should consider that *F. alnus* is present inside the forest in areas that are not currently being reached by strict roadside treatments. Leaving these individuals untreated can lead to reinvasion in the area. This study also indicates that *F. alnus* is spatially aggregated, so we suggest spatially targeted detection and management of *F. alnus*.

## Figures and Tables

**Table 1:** Summary of data collected for the presence and Buckthorn Index (*BI*) values of *F. alnus*.

Site	Number of Sample Points	*BI* ± SEM	% Samples with *F. alnus* (Presence)
1	459	17.1 ± 0.08	83.0
2	394	7.2 ± 0.45	67.8
3	286	30.3 ± 1.54	84.6
4	273	12.8 ± 1.06	64.5

**Table 2:** Parameters of geostatistical analysis for Buckthorn Index (*BI*) values and presence values. The degree of spatial dependency (*DSD*), expressed in percentage, was indicative of the spatial dependency between the sample points at each site. The range column expresses the maximum distance from the road in which *F. alnus* individuals are expected to be autocorrelated with each other. R^2^ and residual sums of squares (RSS) were both optimized to increase the accuracy of our calculations.

Variable	Site	Model	Nugget	Sill	Partial Sill	*DSD* (%)	Range (m)	R^2^	RSS
Presence of *F. alnus* (%)	1	Exponential	0.017	0.141	0.124	87.94	3.7	0.443	0.00113
	2	Nugget	0.182	0.252	0.070	27.78	202.5	0.943	0.00060
	3	Nugget	0.130	0.130	0	0	203.8	0.309	0.00206
	4	Exponential	0.122	0.246	0.124	50.41	31.8	0.942	0.00106
Buckthorn Index (*BI*)	1	Spherical	110.6	369.1	258.5	70.04	155.0	0.993	983
	2	Exponential	61.1	128.1	67.0	52.30	340.8	0.858	287
	3	Exponential	284.0	689.7	405.7	58.82	45.2	0.811	42,461
	4	Exponential	138.6	340.4	201.8	59.28	30.9	0.837	7860

**Table 3:** Parameters and associated statistics of SADIE. *I_a_* represents the index of aggregation, and a value greater than one represents an aggregated spatial pattern. *P*_a_ indicates the significance of the respective *I*_a_ value, where a value of less than 0.05 is significant. *v*_i_ and *v*_j_ are the indices of clustering.

Variable	Site	*I*_a_	*P*_a_	Mean *v*_j_ *	Mean *v*_i_ **	*p* (Mean *v*_j_)	*p* (Mean *v*_i_)
Buckthorn Index (*BI*)	1	5.18	0.0003	−5.065	4.579	<0.0001	<0.0001
	2	3.49	0.0003	−3.213	2.912	<0.0001	<0.0001
	3	1.74	0.0003	−1.649	1.746	0.0015	0.0005
	4	2.64	0.0003	−2.857	2.639	<0.0001	<0.0001
Presence (%)	1	2.40	0.0003	−2.405	2.422	<0.0001	<0.0001
	2	4.13	0.0003	−4.062	4.197	<0.0001	<0.0001
	3	1.91	0.0003	−1.832	1.944	0.0005	<0.0001
	4	1.83	0.0015	−1.904	1.740	0.0008	0.0023

* *v*_i_ = index of clustering for points in which the *BI* or presence value is above average for that site. ** *v*_j_ = index of clustering for points in which the *BI* or presence value is below average for that site.

## Data Availability

Data will be available upon request.

## Supplementary Materials

The following supporting information can be downloaded at: https://www.mdpi.com/article/10.3390/biology12111393/s1, Table S1. Parameters of anisotropic semivariograms for Buckthorn Index (*BI*) and percent presence of *F. alnus.*
