# Supplementary material for: Spatial Patterns of Frangula alnus (Rosales: Rhamnaceae): Implications for Invasive Plant Management
Source: Biology (Basel). 2023 Nov 1;12(11):1393. doi: 10.3390/biology12111393 (PMC10669815; doi:10.3390/biology12111393)
Supplement: Supplementary file 1 [file biology-12-01393-s001.zip › biology-2666306-supplementary.pdf]

Table S1. Parameters of anisotropic semivariograms for Buckthorn Index (*BI*) and percent presence of *F. alnus.*

Variable	Site	Model	Nugget	Sill	Major Range (m)	Minor Range (m)	Anisotropy Factor	R^2	RSS *
Buckthorn Index (*BI*)	1	Spherical	114.8	609.3	410.7	256.1	0.62	0.88	103558
	2	Nugget	66.4	170.6	1960.0	1022.0	0.52	0.26	8625
	3	Exponential	472.4	1383.4	735.0	735.0	1	0.60	732064
	4	Exponential	201.2	637.5	751.0	751.0	1	0.66	107808
Presence of *F. alnus* (%)	1	Nugget	0.124	0.332	867.2	668.5	0.77	0.44	0.00094
	2	Nugget	0.164	0.435	527.6	527.6	1	0.94	0.05310
	3	Gaussian	0.107	0.608	754.6	754.6	1	0.48	0.3070
	4	Nugget	0.179	0.451	499.2	499.2	1	0.42	0.0464

* RSS: Residual sum of squares
